# Formulation of Quaternized Aminated Chitosan Nanoparticles for Efficient Encapsulation and Slow Release of Curcumin

**DOI:** 10.3390/molecules26020449

**Published:** 2021-01-16

**Authors:** Ahmed M. Omer, Zyta M. Ziora, Tamer M. Tamer, Randa E. Khalifa, Mohamed A. Hassan, Mohamed S. Mohy-Eldin, Mark A. T. Blaskovich

**Affiliations:** 1 Polymer Materials Research Department, Advanced Technology and New Materials Research Institute (ATNMRI), City of Scientific Research and Technological Applications (SRTA-City), New Borg El-Arab City, Alexandria 21934, Egypt; ttamer85@gmail.com (T.M.T.); randaghonim@yahoo.com (R.E.K.); mmohyeldin@srtacity.sci.eg (M.S.M.-E.); 2 Centre for Superbug Solutions, Institute for Molecular Bioscience, The University of Queensland, Brisbane, QLD 4072, Australia; z.ziora@uq.edu.au (Z.M.Z.); m.blaskovich@uq.edu.au (M.A.T.B.); 3 Protein Research Department, Genetic Engineering and Biotechnology Research Institute (GEBRI), City of Scientific Research and Technological Applications (SRTA-City), New Borg El-Arab City, Alexandria 21934, Egypt; madel@srtacity.sci.eg

**Keywords:** nanoparticles, quaternized aminated chitosan, curcumin slow release

## Abstract

An effective drug nanocarrier was developed on the basis of a quaternized aminated chitosan (Q-AmCs) derivative for the efficient encapsulation and slow release of the curcumin (Cur)-drug. A simple ionic gelation method was conducted to formulate Q-AmCs nanoparticles (NPs), using different ratios of sodium tripolyphosphate (TPP) as an ionic crosslinker. Various characterization tools were employed to investigate the structure, surface morphology, and thermal properties of the formulated nanoparticles. The formulated Q-AmCs NPs displayed a smaller particle size of 162 ± 9.10 nm, and higher surface positive charges, with a maximum potential of +48.3 mV, compared to native aminated chitosan (AmCs) NPs (231 ± 7.14 nm, +32.8 mV). The Cur-drug encapsulation efficiency was greatly improved and reached a maximum value of 94.4 ± 0.91%, compared to 75.0 ± 1.13% for AmCs NPs. Moreover, the in vitro Cur-release profile was investigated under the conditions of simulated gastric fluid [SGF; pH 1.2] and simulated colon fluid [SCF; pH 7.4]. For Q-AmCs NPs, the Cur-release rate was meaningfully decreased, and recorded a cumulative release value of 54.0% at pH 7.4, compared to 73.0% for AmCs NPs. The formulated nanoparticles exhibited acceptable biocompatibility and biodegradability. These findings emphasize that Q-AmCs NPs have an outstanding potential for the delivery and slow release of anticancer drugs.

## 1. Introduction

Drug delivery systems (DDSs) are pharmaceutical formula or devices designed to overcome the present limitations of conventional therapeutics [1]. DDSs have the ability to improve the biological activity, enhance the therapeutic index or extend the biological half-life of drugs [2,3]. Various materials including microspheres, mesoporous, micro/nanoparticles, liposomes, micelles, and emulsions were developed as effective drug carriers [4]. Currently, a wide range of nanoparticles are being advanced to serve as potential carriers for delivering drugs in a controlled mode to specific-targeted sites in the human body [5,6,7,8]. These nano-sized systems have a remarkable aptitude for the targeted delivery of a specific drug dose to specific cells (such as cancer cells), without disturbing the physiology of the normal cells [9]. In addition, the targeted nano systems are able to ameliorate poor drug diffusion/release profiles, improve drug solubility, and boost bio-distribution and immunogenicity [10].

Chitosan (Cs), a natural biopolymer, is derived from chitin, which is the main component for the exoskeleton of crabs, shrimps, and lobsters [11]. Chitosan is composed of β-(1,4)-2-acetamido-d-glucose and β-(1,4)-2-amino-d-glucose groups, and possesses a polycationic character. Due to its intriguing bio-characteristics, such as biocompatibility, biodegradability, non-toxicity, and bioavailability [12], Cs are selected as an ideal candidate for several medical and pharmaceutical applications, including wound dressing, tissue engineering, and drug delivery systems [13,14]. Furthermore, Cs have the capability to form gel-nanoparticles via ionic crosslinking with polyanionic molecules, such as sodium tri-polyphosphate (TPP) [15]. Cs are also established as a nanoscopic carrier for diverse drugs, with chitosan-based nanoparticles (NPs) evolving to be one of the most accepted delivery systems for cancer chemotherapy [16,17]. Numerous chemical modifications, including the Schiff base formation [18], grafting [19], and quaternization [20], were conducted on native chitosan to broaden the spectrum of its biological activities. For instance, amine-functionalized chitosan (aminated chitosan; AmCs) is a newly developed chitosan derivative with extra amine groups, which displays better anti-microbial activity, decent blood biocompatibility, and biodegradability [21]. Consequently, an AmCs derivative was been successfully employed as an effective candidate for advanced wound dressing and oral drug delivery systems [22,23].

Recently, close attention was paid to the modified water-soluble forms of chitosan derivatives, since their improved characteristics can play an important role in the pharmaceutical fields [24]. Among these modifications, quaternization of Cs can significantly overcome the poor solubility of chitosan at a neutral pH, and consequently, widen its possible applicability for gene/drug delivery, tissue engineering, and wound dressing [25,26]. Therefore, quaternized chitosan (Q-Cs) derivatives are able to enhance the diffusion of drug molecules through various biological barriers in neutral/alkaline conditions [27]. These Q-Cs derivatives expose permanent positive charges that positively affect the mucoadhesivity, biocompatibility, controlled biodegradability, and drug loading/release profiles [28]. Several Q-Cs derivatives such as trimethyl-chitosan (TMC), triethyl-chitosan (TEC), dimethylethyl-chitosan (DMEC), and *N*-[(2-hydroxy-3-trimethylammonium) propyl] chitosan chloride (HTCC) are established as potential carriers for protein, vaccines, and anti-cancer drugs [29,30,31,32].

Herein, we aimed to combine the beneficial features of the tuned size and surface characteristics of nanocarriers, with the attractive bio-characteristics of quaternized chitosan derivatives. Thus, new nanoparticles-based quaternized aminated chitosan (Q-AmCs) derivatives were formulated for the oral delivery and slow release of Curcumin (Cur; anti-cancer drug compound), at the site of the colon. The developed nanoparticles were examined to determine their structures, thermal properties, and morphological changes through Fourier transform infrared spectroscopy (FT-IR), thermogravimetric analyzer (TGA), and transmission electron microscope (TEM). In addition, particle size and surface charges of the formulated nanoparticles were also determined. Parameters affecting the nanoparticles formation as well as Cur-drug encapsulation efficiency were optimized. Effects of the nanoparticles compositions and drug amount on the in vitro drug release profiles were also explored. Finally, biocompatibility and biodegradability of the formulated nanoparticles under the physiological environments were also evaluated.

## 2. Results and Discussion

### 2.1. FT-IR Analysis

**Figure 1:**
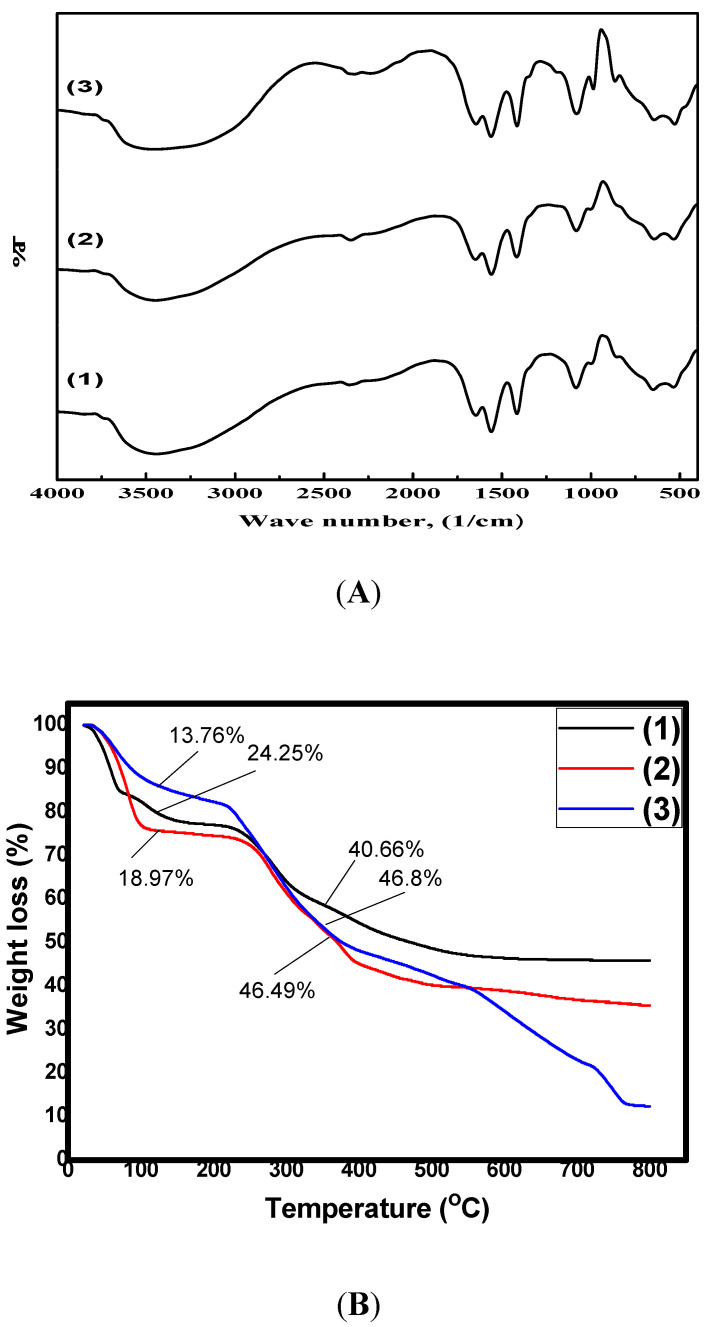
(**A**) FT-IR spectra and (**B**) TGA thermograms of (1) AmCs, (2) Q-AmCs1, and (3) Q-AmCs2 nanoparticles.

The FT-IR spectra of the formulated nanoparticles were collected to provide additional information regarding their chemical structures, as presented in Figure 1A. It was clear that the basic characteristics of the chitosan polysaccharide structure were retained at 852–1170 cm^−1^. The observed characteristics included a broad band at around 3441 cm^−1^, in the AmCs spectrum that corresponded to the stretching vibrations of amine and hydroxyl groups [33]. These bands were shifted to 3446 and 3452 cm^−1^ in Q-AmCs1 and Q-AmCs2 derivatives. In addition, the bands at 1645–1649 cm^−1^ and 1560 cm^−1^ were assigned to the stretching vibration of the amide-I (C=O of NH-C=O) groups and the N-H bending of amide-II. Furthermore, the observed multiple-peaks in the wave number range of 986–1186 cm^−1^ were associated with the existence of the C-C, C-O, and C-O-C glycoside bonds. The FT-IR spectra of Q-AmCs derivatives displayed characteristic absorption bands at 1855 and 1003 cm^−1^, which could be ascribed to the C-N stretching vibration of the quaternary ammonium groups. Likewise, the peak intensities increased with increasing 2-chloro-*N*,*N*-diethylethylamine hydrochloride ratio (Q-AmCs2) in the reaction mixture, confirming the successful formation of the Q-AmCs. Finally, the absorption bands observed at around 1380–1400 cm^−1^ were assigned to the C-H symmetric bending of the CH_3_ groups on the quaternary ammonium groups [34].

### 2.2. Thermal Analysis

The thermogravimetric analyzer (TGA) was applied to investigate the thermal stabilities of the AmCs and Q-AmCs nanoparticles with rising temperature, up to 800 °C. Figure 1B illustrates that the thermal degradation profiles for the formulated nanoparticles occurred in three sequential stages. The first stage was observed at the ambient temperature (0–120 °C), with recorded maximum weight losses of 24.25, 18.97, and 13.76% for AmCs, Q-AmCs1, and Q-AmCs2, respectively. This initial weight loss was caused by the release of the moisture content from the tested samples, including water bound by the hydrophilic groups of the nanoparticle matrix [35]. The second stage was detected at elevated temperatures up to 350 °C, and was assigned to the cleavage of the C-O-C glycoside bonds and decomposition of the pyranose ring of AmCs and its quaternized forms. Finally, the third depression stage (beyond 400 °C) could be attributed to the thermal decomposition of adducts. These decomposition stages were consistent with the reported main thermal decomposition process of nanoparticle based-chitosan polysaccharide and its derivatives [22]. Table 1 summarizes that Q-AmCs1 and Q-AmCs2 needed temperature increases up to 370.51 and 376.9 °C, for loss of half their weights (T_50%_ °C) compared to 475.5 °C, for the AmCs sample. It could be concluded from the obtained TGA data that the thermal stability of the AmCs nanoparticles was higher than its quaternized forms.

### 2.3. Morphological Analysis

**Figure 2:**
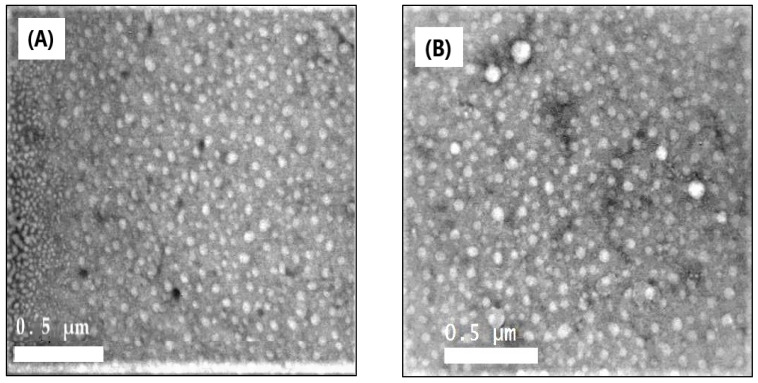
TEM images of (**A**) AmCs and (**B**) Q-AmCs2 nanoparticles.

Figure 2A,B illustrate the TEM images of the formulated AmCs and Q-AmCs nanoparticles. The images clarify that both the AmCs and its quaternized nanoparticles were well-dispersed, uniform in their size, and almost spherical in their shapes, except for a few pentagonal structures [36]. Moreover, the quaternization process slightly affected the morphology of the nanoparticles, since few large particles were noticed in the TEM image of Q-AmCs, as a result of the polarity difference between the compositions of nanoparticles (AmCs and 2-chloro-*N*,*N*-diethylethylamine hydrochloride).

### 2.4. Zeta Potentials and Particle Size Evaluation

**Figure 3:**
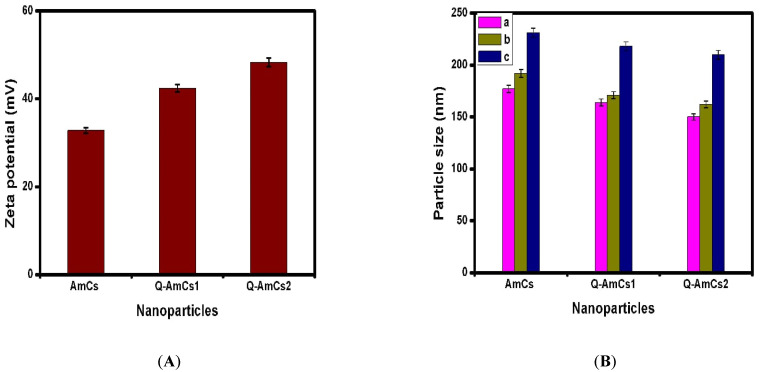
(**A**) Zeta potential and (**B**) particle size values of the formulated nanoparticles at different polymer/TPP ratios of (a) 5:1, (b) 10:1, and (c) 10:1.5. All measurements were performed in triplicates (*n* = 3), and values were expressed as mean + standard deviation (±SD).

The zeta potential of the formulated nanoparticles was measured, as shown in Figure 3A. The results revealed that the AmCs and Q-AmCs nanoparticles had high positive charges on their entire surfaces. In addition, the Q-AmCs1 and Q-AmCs2 recorded higher values of +42.4 and +48.3 mV, compared to +32.8 mV for AmCs nanoparticles. These results confirmed that quaternization of AmCs increased the positive charges on the quaternized form derivative, as a result of the formation of positively charged quaternal ammonium salts. As expected, increasing the quaternization ratio significantly augmented the zeta potential values, since more positive charges were present after formation of the nanoparticles. According to the previous studies, the nanoparticles could be present in a stable state when their surface potentials were more than 30 mV, as a result of enhancing the electrostatic repulsion forces between similar positive charges [37]. Additionally, increasing the surface positive charges promoted the electrostatic attraction forces with the negatively charged particles on the cell membrane.

Additionally, at a constant polymer ratio, the increasing TPP ratio (i.e., from 10:1 to 10:1.5) resulted in an increase in the nanoparticle size, as presented in Figure 3B, with values of 231 ± 7.14, 218 ± 4.91, and 210 ± 9.40 nm for AmCs, Q-AmCs1, and Q-AmCs2 nanoparticles, respectively. It was also observed that increasing the AmCs (or QAmCs) ratio with a constant TPP ratio (i.e., from 5:1 to 10:1) slightly increased the particle size from 177 ± 11.24 to 192 ± 10.22 nm (for AmCs), from 164 ± 6.54 to 171 ± 8.35 nm (for Q-AmCs1), and from 150 ± 5.44 to 162 ± 9.10 nm (for Q-AmCs2) nanoparticles, respectively. These results could be rationalized, based on the hypothesis that increasing the polymer ratio led to increasing the polymer solution viscosity, which directly promoted the ionic gelation process with more adhesion. Thus, deposition of some nanoparticles took place, resulting in formation of larger nanoparticles. Moreover, increasing the TPP and AmCs (or Q-AmCs) ratios could possibly generate more electrostatic attraction forces between the negatively charged TPP and the polycation polymer (AmCs and Q-AmCs), and thus, the particle size increased consequently. The results denoted that the quaternized AmCs nanoparticles displayed smaller particle size (150 ± 5.44–218 ± 4.91 nm) compared to the native AmCs nanoparticles (177 ± 11.24–231 ± 7.14 nm), and the size decreased with rising quaternization ratio in the nanoparticles matrix. This behavior was noticed for all tested polymer/TPP ratios. It was stated that the effective particle size for the injectable drug nanocarriers was mostly below 200 nm [38,39]. These sizes were met by our results for all formulated nanoparticles, with polymer/TPP ratios of 5:1 and 10:1, and thus, they were selected for the subsequent encapsulation studies.

### 2.5. Cur-Drug Loading Evaluation

**Figure 4:**
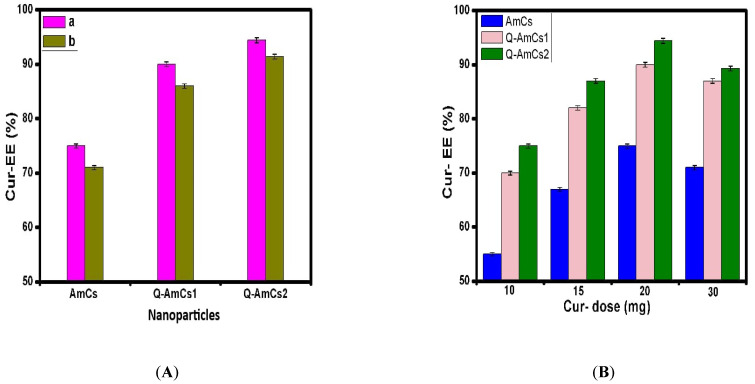
Encapsulation efficiency (EE%) values of the Cur-loaded NPs at (**A**) different polymer/TPP ratios [(a) 5:1and (b) 10:1], and (**B**) at different initial Cur-dose. All measurements were performed in triplicates (*n* = 3), and the values were expressed as mean + standard deviation (±SD).

Considerable loading efficiency was required to attain the anticipated therapeutic impact of the drug dose, without hampering its biological action. Herein, the Curcumin-drug was successfully loaded into the formulated nanoparticles, with the encapsulation efficiency shown in Figure 4. At a constant Cur-drug amount (20 mg), the Cur-encapsulation efficiency was greatly improved after the quaternization process, with the highest value of 94.4 ± 0.91% recorded using the Q-AmCs2 nanoparticles, compared to 75 ± 1.13% for AmCs nanoparticles. In addition, a slight decrease in the encapsulation efficiency value was noticed when increasing the polymer/TPP ratio from 5:1 to 10:1 (Figure 4A). These results were consistent with improving the positively charged surface of nanoparticles with the increasing quaternization ratio, and agreed with the zeta potential measurements. Thus, more Cur-drug molecules were able to bind with the positive Q-AmCs surface via several interactions, comprising hydrogen bonding and the electrostatic attractions between the opposite charges [40]. This in turn led to enhancing the encapsulation efficiency values. In contrast, raising the polymer ratio (AmCs or Q-AmCs) at a constant TPP ratio (i.e., 10:1) would produce a viscous solution that adversely affected the dispersion of drug molecules through the polymer matrix, resulting in a slight decrease in the encapsulation efficiency.

Figure 4B illustrates that as the initial Cur-dose increased, the encapsulation efficiency increased in general. For all tested nanoparticles, the Cur-EE% values were greatly augmented with increasing the dose from 10 to 20 mg. These results could be related to increasing the probabilities of more Cur-drug molecules to be encapsulated by the formulated nanoparticles. However, upon further increase in the Cur-drug dose up to 30 mg, the Cur-EE% decreased from 75 ± 1.13 to 71 ± 0.743% (for AmCs NPs), from 90 ± 0.62 to 87 ± 0.61% (for Q-AmCs1 NPs), and from 94.4 ± 0.91 to 89.3 ± 0.55% (for Q-AmCs2 NPs), respectively. This reduction could be a result of the limited capacity of the nanoparticles, which might have reached the saturation state, by using 20 mg of Cur-drug. The higher initial drug dose (30 mg) could hinder the passage as well as obstruct the proper dispersion of Cur-molecules through the polymer matrix. Thus, some of drug molecules were not encapsulated, and they mostly leached through the formulation process.

### 2.6. In Vitro Cur-Drug Release Evaluation

**Figure 5:**
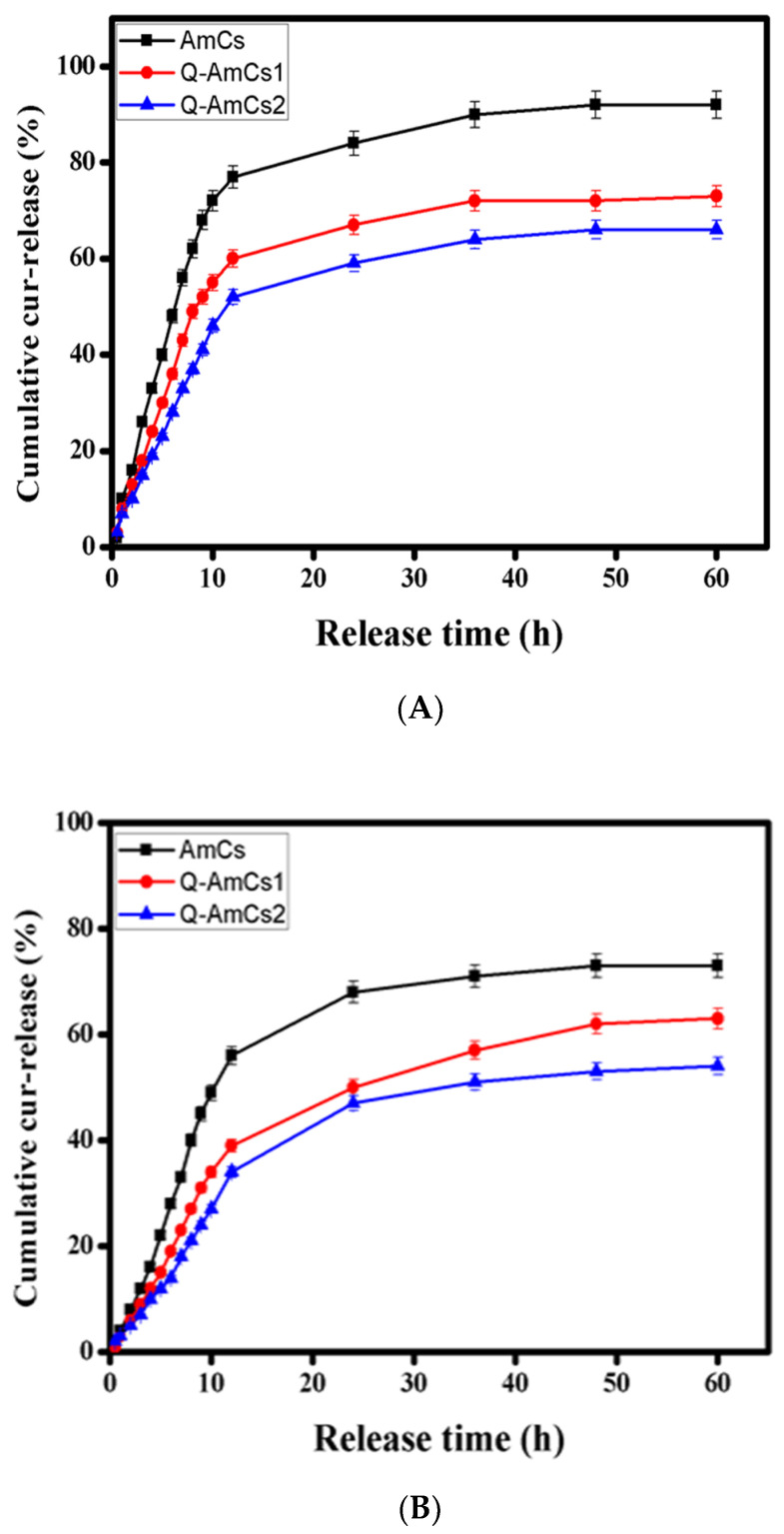
Cumulative Cur-release profiles of the formulated Cur-loaded NPs at (**A**) SGF (pH 1.2) and (**B**) SCF (pH 7.4). All measurements were performed in triplicates (*n* = 3), and the values were expressed as mean + standard deviation (±SD).

The release profiles of Cur-drug were investigated under simulated gastrointestinal conditions, and the results are depicted in Figure 5A,B. The results showed a rapid Cur-release from all tested nanoparticles at the initial release stage. It was observed that after 12 h from the initial release time, the maximum cumulative release values were recorded for AmCs, Q-AmCs1, and Q-AmCs2, respectively, as—77.0, 60.0, and 52.0% at pH 1.2, and 56.0, 39.0, and 34.0%, at pH 7.4. Subsequently, a slow release rate was noticed with the prolongation of the Cur-release time for all formulations. For the Cur-loaded Q-AmCs NPs, the in vitro Cur-release rate was greatly decreased at pH 1.2 [SGF] and pH 7.4 [SCF], compared to that of Cur-loaded AmCs NPs. The results also showed that increasing the quaternization ratio in the Q-AmCs NPs led to a further decrease in the Cur-release values. After 60 h, the cumulative Cur-release values at pH 1.2 were decreased from 73.0% (Q-AmCs1) to 66.0% (Q-AmCs2), and from 63.0% (Q-AmCs1) to 54.0% (Q-AmCs2) at pH 7.4. Cur-loaded AmCs NPs recorded the highest cumulative release values of 92.0 and 73.0% at pH 1.2 and pH 7.4, respectively. Similar results were obtained by Sun et al. [41]. They reported that a sustained Curcumin release took place from the nanoparticles based on the Ion-Crosslinking Aminochitosan-modified folic acid (FA). The cumulative release (%) of Curcumin reached a maximum value of 56.2% after 48 h, compared to 54% after 60 h in the present study. Hasan et al. observed that the Curcumin release was much lower in the case of chitosan-coated nanoliposomes compared to the uncoated nanoliposomes [42]. Additionally, Nasab et al. indicated that the existence of chitosan shell on the surface of mesoporous silica nanocarriers greatly improved the slow and sustained release rate of Curcumin at a low pH, compared to the environment pH [43].

In fact, most of the NH_2_ groups of AmCs NPs were consumed through the ionic crosslinking with TPP for the formation of nanoparticles, while the remaining free groups would be protonated at pH 1.2 and transformed into positively charged NH_3_^+^ groups. These positive charges could easily interact with the negative charges of the Cur-drug via electrostatic attraction forces, resulting in a decrease in the cumulative release rate. Furthermore, the introduced quaternary ammonium groups provide permanent positive charges in the Q-AmCs NPs, as verified by the zeta potential measurements. This would directly generate very strong electrostatic interactions with the Cur-drug, which would further prevent its rapid release, and thus, an additional decline for the cumulative release rate took place. These results presented in Figure 5 were in the agreement with other reported studies [25,44].

### 2.7. Biocompatibility and Biodegradability Evaluation

**Figure 6:**
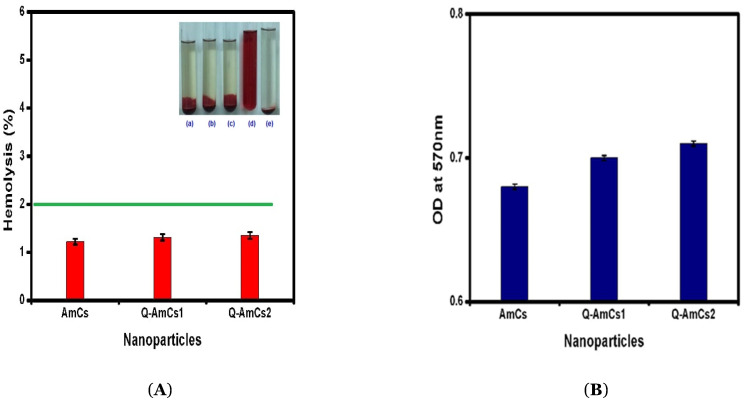
(**A**) Hemocomptibility data and blood-containing tubes images of (a) AmCs NPs, (b) Q-AmCs1 NPs, (c) Q-AmCs2 NPs, (d) positive control, and (e) negative control) nanoparticles. (**B**) Biodegradation profiles of AmCs, Q-AmCs1, and Q-AmCs2 NPs shown by the formation of DNS adduct with liberated sugars, after incubation of NPs (10 mg) with lysozyme for 3 days, at 37 °C. All measurements were performed in triplicates (*n* = 3), and the values were expressed as mean + standard deviation (±SD).

According to the standard protocol for testing the hemolytic properties of materials (ASTM F756-00), biomaterials could be characterized as hemolytic materials (hemolytic index over 5%), slightly hemolytic materials (hemolytic index in the range of 2–5%), and non-hemolytic materials (hemolytic index in the range of 0–2%) [45]. Figure 6A displayed the blood-containing tube images and data of hemocompatibility for the formulated nanoparticles, which was evaluated as a function of the blood-hemolysis percent. The data demonstrated that the as-formulated AmCs and Q-AmCs nanoparticles showed little hemolysis. The good hemocompatibility could be attributed to the excellent biocompatibility nature of chitosan biopolymer. Figure 6B represents the biodegradation profiles of the developed nanocarriers, measured by incubating the lysozyme solution. The glycosidic bonds could be hydrolyzed by the lysozyme enzyme, resulting in a biodegradation of the polysaccharide structure. This enzymatic activity might be enhanced by the hydrophilic groups in the nanoparticles matrix, which would potentially allow for adsorption of enzyme. The results showed that all tested samples were biodegraded by the lysozyme enzyme, due to their core construction [46,47].

## 3. Materials and Methods

### 3.1. Materials

Chitin from shrimp shells (>95% acetylated), 2-chloro-*N*,*N*-diethylethylamine hydrochloride (assay 99%), Curcumin (Cur; ≥94% curcuminoid content), ethylenediamine (EDA; assay ≥99%), *N*-methyl-2-pyrrolidinone (NMP; purity 98%), sodium tripolyphosphate (TPP; assay 85%), sodium iodide (NaI; assay ≥99%), and para-benzoquinone (PBQ; assay ≥94%) were purchased from Sigma-Aldrich Chemie GmbH (Taufkirchen, Germany). Sodium hydroxide (pellets; assay 98%), acetic acid (purity 98%), acetone (assay 99%), and ethanol (assay 99%) were delivered by El-Nasr Pharmaceutical Co. (Cairo, Egypt). All chemicals and solvent were of analytical grade and were used without further purification.

### 3.2. Synthesis of AmCs Derivative

The aminated chitosan derivative was prepared and purified according to the authors’ previous work [21,48]. In brief, an accurate amount of chitin (8 g) was dispersed in the PBQ (6.9 mM; 100 mL) solution, under gentle stirring. The reaction was conducted at room temperature for 6 h, while the pH of the mixture was raised to 10 using the NaOH (0.1 mol/L) solution. Then, the mixture content was filtered and washed with distilled water to eliminate the unreacted PBQ molecules. The resultant activated chitin was then soaked at room temperature, in a solution of EDA (1.8 mM; 100 mL) for further 6 h, to ensure the complete amination step. The produced aminated chitin was separated and washed several times by distilled water, to remove the excess EDA molecules. Finally, the resultant aminated chitin was immersed in NaOH (50%) under a reflux conditions (12 h; 120 °C) to complete the deacetylation process. The obtained aminated chitosan (AmCs) was separated from the reaction medium and followed by filtration, washing until neutrality and drying in a vacuum oven at 60 °C.

### 3.3. Synthesis of Q-AmCs Derivative

**Figure 7:**
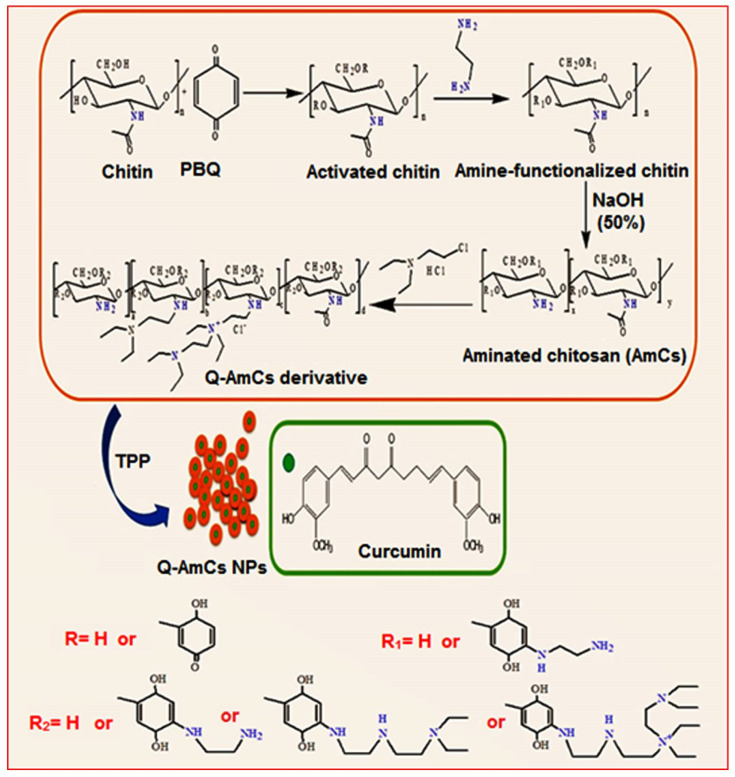
A schematic diagram for the synthesis of quaternized aminated chitosan (Q-AmCs) derivative.

Quaternization of AmCs derivative was achieved according to the reported reductive alkylation method [49], with a slight modification. In a 2-necked flask, AmCs (1 g) and sodium iodide (4.8 g; as a catalyst) were dissolved in 80 mL of *N*-methyl-2-pyrrolidinone/acetic acid (1%), using shaking water bath at 60 °C, until it reached complete dissolution of AmCs. Next, the flask was connected to a condensation column, followed by the addition of excess of 2-Chlorotriethylamine hydrochloride solution under continuous stirring, while NaOH (15% (*w*/*v*); 11 mL) was added dropwise during the reaction process. The product was then precipitated using 200 mL of ethanol, centrifuged, washed by acetone, and finally dried under reduced pressure. Two ratios of 2-chloro, *N*,*N*-diethylethylamine hydrochloride were used per 1 mole of AmCs and coded as: (Q-AmCs1 [1:0.534 M], and Q-AmCs2 [1:1.602 M]. The proposed reaction mechanism for the preparation of AmCs and Q-AmCs derivatives is presented in Figure 7.

### 3.4. Formulation of Q-AmCs NPs

Q-AmCs NPs were formulated according to the ionic gelation technique, using TPP as an ionic crosslinker [31]. First, an exact quantity of the Q-AmCs powder was dissolved at room temperature in de-ionized water, with a final concentration of (0.2%; *w*/*v*). Next, the TPP solution (0.1%; *w*/*v*) was dropped slowly into the prepared Q-AmCs solution via a peristaltic pump, under high stirring speed at room temperature. After 20 min, the formed nanoparticles were separated from the gelling medium through centrifugation of the dispersion at 12,000 rpm for 20 min. The collected nanoparticles were freeze-dried overnight and followed by sortation at 4 °C. Three ratios of Q-AmCs/TPP were used as 5:1, 10:1, and 10:1.5. Additionally, the same steps were applied for the formulation of AmCs nanoparticles, except for dissolving AmCs (0.2%; *w*/*v*) in acetic acid (1%; *w*/*v*) instead of the de-ionized water.

### 3.5. Cur-Drug Loading Step

To encapsulate the Cur-drug into the developed Q-AmCs NPs, a precise amount of Cur-drug (10–30 mg) was dissolved in 5 mL of ethanol, and subsequently added to the previously prepared AmCs and Q-AmCs solutions, with continuous stirring for 30 min at room temperature, to obtain homogenous mixtures. The same procedure described above (Section 3.4) was followed to obtain Cur-loaded Q-AmCs nanoparticles. In order to assess the Cur-encapsulation efficiency (Cur-EE (%)), the freeze-dried Cur-loaded Q-AmCs NPs were re-suspended in ethanol, twisted using Vortex, and centrifuged. By using a UV-spectrophotometer, the leached amount of Cur-drug from nanoparticles was estimated at 429 nm [44], and the Cur-EE (%) was calculated according to Equation (1). The filtrate solution of free drug–nanoparticle was used as a control reference.

(1) $$\mathrm{Cur}-\mathrm{EE}\;\left(\%\right)=\frac{W_{i}-W_{f}}{W_{i}}\times 100$$

where *W_i_* represents the initial quantity of the Cur-drug added to the Q-AmCs solution, and *W_f_* is the final quantity of Cur-drug per weighted amount of the loaded NPs.

### 3.6. Physicochemical Characterization

The functional groups of the developed nanoparticles were determined by Fourier transform infrared spectroscopy (FT-IR, Model 8400 S, Shimadzu, Kyoto, Japan) and thermal examination was done by Thermogravimetric analyzer (TGA, Model 50/50H, Shimadzu). The morphological changes were investigated by a Transmission electron microscope (TEM, JEM-100CX, JEOL INC., Peabody, Kansas, USA). The average sizes of the formulated nanoparticles were estimated by a Particle size analyzer (Beckman, Coulter N5, Brea, CA., USA). Additionally, determination of the surface charges was achieved by the Zeta-Sizer (Malvern Panalytical Co., Royston, UK).

### 3.7. In Vitro Cur-Drug Release Study

The formulated Cur-loaded nanoparticles were examined for the Cur-drug release efficiency under simulated intestinal conditions. Herein, a definite amount of tested samples was re-dispersed in separate aqueous solutions of saline phosphate buffer (pH 7.4; 10 mL) as a simulated colon fluid [SCF], and HCl (pH 1.2; 10 mL) as a simulated gastric fluid [SGF]. The in vitro release process was performed by a dissolutor instrument under constant conditions (50 rpm, 37 °C). After time intervals, 1 mL of the release medium was collected for assaying; while an equivalent volume of the freshly prepared buffer was added to the release medium. The cumulative Cur-release percentage was measured by a UV-spectrophotometer at 429 nm.

### 3.8. Biocompatibility Test

Biocompatibility of the formulated nanoparticles was investigated according to the previously reported method, with a minor modification [45]. A known amount of freeze-dried nanoparticles (10 mg) was first washed using a fresh phosphate buffer (pH 7.4) solution for 72 h. Next, the tested samples were rinsed and soaked in a glass test tube containing an ACD-blood mixture, which was previously prepared by mixing fresh human blood (9 mL) with acid citrate dextrose (1 mL; as anti-coagulant). For blood tests, informed consent was obtained from a volunteer before the use of his blood and the examinations were carried out in compliance with relevant guidelines. The mixtures were maintained at 37 °C for 3 h, followed by centrifugation for 15 min at 20,000 rpm. A control was performed using a free-sample mixture, while phosphate buffer and water were used as negative and positive controls, respectively. The supernatants were examined via estimation of the optical density (OD) at 540 nm, using a UV-spectrophotometer, and their hemolysis percent was calculated according to the following equation:

(2) $$\mathrm{Hemolysis}\;\left(\%\right)\;=\;\frac{\left(\mathrm{OD}\mathrm{sample}\;-\;\mathrm{OD}\mathrm{negative}\;\mathrm{control}\right)}{\left(\mathrm{OD}\mathrm{positive}\;\mathrm{control}\;-\;\mathrm{OD}\mathrm{negative}\;\mathrm{control}\right)}\times \;100$$

### 3.9. In Vitro Biodegradability Test

Biodegradability of the formulated nanoparticles was examined according to the previously published method [50]. A known quantity of tested nanoparticles (10 mg) was soaked for 3 days at 37 °C, in a tube containing a mixture of phosphate buffer (2 mL; pH 7.0) and lysozyme solution (0.5 mL). The activity of lysozyme was stopped by the addition of 3,5-dinitrosalicylic acid (DNS; 1.5 mL) used as a reagent for the estimation of reduced sugar from the nanoparticle matrix. Subsequently, the mixture was transferred into a water bath, left for boiling at 100 °C for 15 min, and followed by cooling at room temperature. The produced color from the reaction of the reduced liberated sugar from nanoparticles, with the DNS-reagent, was analyzed by measuring the optical density (OD) at a wavelength of 570 nm using a visible-spectrophotometer.

All experiments were performed in triplicates (*n* = 3), and the gained data were expressed as the mean + standard deviation (±S.D.)

## 4. Conclusions

In this study, quaternized aminated chitosan nanoparticles were formulated and characterized. The developed nanoparticles showed acceptable particle size (162 ± 9.10 nm) and a higher surface potential (+48.3 mV) compared to the unmodified AmCs. The results signified that Q-AmCs NPs could encapsulate 94.4 ± 0.91% of the Cur-drug (20 mg), as compared to 75.0 ± 1.13% in the AmCs NPs. The release studies confirmed that the Cur-release rate was decreased at pH 7.4 [SCF], with an increasing quaternization ratio, and recorded a maximum value of 54%, after 60 h of the initial release time. Bioevaluation studies clarified that the formulated NPs were biocompatible (non-hemolytic) and biodegradable, under the physiological conditions. Therefore, the Q-AmCs NPs could be potentially used for the delivery and slow release of anticancer drugs.

## Figures and Tables

**Table 1:** TGA data for AmCs and Q-AmCs nanoparticles.

Nanoparticles	Weight Loss (%)		T_50%_ °C
	Ambient 0–120 °C	Up to 350 °C	
AmCs	24.25	40.66	475.50
Q-AmCs1	18.97	46.8	370.51
Q-AmCs2	13.67	46.49	376.9

## Data Availability

The data presented in this study are available on request from the corresponding author.

## Sample Availability

Samples of the compounds are available from the authors.
